# Assessing access to cardiologists and endocrinologists in the Texas ACA market

**DOI:** 10.1093/haschl/qxag068

**Published:** 2026-03-21

**Authors:** Simon F Haeder, Yuqin Lou, Wendy Xu

**Affiliations:** Division of Health Services Management & Policy, College of Public Health, The Ohio State University, Columbus, OH 43210, United States; Division of Health Services Management & Policy, College of Public Health, The Ohio State University, Columbus, OH 43210, United States; Division of Health Services Management & Policy, College of Public Health, The Ohio State University, Columbus, OH 43210, United States

**Keywords:** managed care, Affordable Care Act, Texas, cardiology, endocrinology, provider networks, provider directories, access to care

## Introduction

Access to specialists such as cardiologists^1^ and endocrinologists^2^ is an important part of comprehensive medical care. However, evidence suggests that Americans cannot always access care when needed.^3^ In a managed-care setting inaccurate provider directories and inadequate provider networks may be particularly important factors limiting access.^3,4^ The degree to which this is the case has been explored in only a limited way, particularly in the context of cardiologists and endocrinologists or Affordable Care Act (ACA) marketplaces, which provide coverage to more than 4 million Texans and more than 24 million Americans.^5^ More generally, both specialties have only seen limited assessment via secret-shopper surveys. Importantly, the 2 specialties are vastly different in terms of provider quantity and distribution, with many fewer practicing endocrinologists and higher degrees of centralization, with likely implications for patient access.^6^

## Data and methods

To assess barriers to cardiologists and endocrinologists, we fielded a secret-shopper survey focused on ACA marketplace coverage in Texas from September 1, 2023, to November 27, 2023, making 3740 calls to cardiologists and 3633 calls to endocrinologists across all ACA marketplace insurers. Calls were proportional to enrollment by pricing region and equally distributed among carriers within each region. We subsequently excluded 2 of the 15 carriers because of the low number of calls. Callers were assigned detailed information about a simulated patient including a home address and insurance coverage. Callers went to the assigned insurer's online provider directory to search for an in-network provider and then called to seek an appointment. Callers indicated that the patient was instructed by their primary care provider to seek care from a specialist.^7^ To assess access for Spanish-speaking patients, we split calls from Hispanic callers evenly between patients seeking care from English-speaking or Spanish-speaking providers. In line with previous work, callers ended calls when put on hold in excess of 5 minutes at a time.^8^

Data were analyzed using used ordinary least squares (OLS) regression or linear probability models with robust SEs, as appropriate, and indicators for rurality of patient and provider as well as insurer. Models were separately estimated for each specialty. Robustness checks with alternative methodologies and specifications provided similar results. Call outcomes presented here were assessed based on the full universe of calls.

## Results

### Cardiologists

Callers generally struggled to reach providers, with 37.3% (95% CI: 36.0% to 38.6%) failing to connect to an individual to verify cardiologists (Table 1). Failure-to-connect rates ranged from 31.7% to 46.4% across carriers (*P* = .037). Verification issues particularly affected rural cardiology callers (36.2% to 40.2%; *P* = .045).

**Table 1.** Overview of unsuccessful and successful calls.

Variable	Failure to connect, % of calls		Any directory errors, % of calls		Capacity limitations, % of calls		Appointments, % of calls		Wait time to appointment, d		Travel time, min	
	Cardio	Endo	Cardio	Endo	Cardio	Endo	Cardio	Endo	Cardio	Endo	Cardio	Endo
Overall	37.3	42.5	27.3	25.2	6.5	7.4	22.8	20.0	31.7	55.2	30.0	39.7
Non-rural patient	36.2	42.5	27.2	25.0	6.7	7.7	24.6	19.8	31.2	50.6	26.9	32.2
Rural patient	40.2	42.4	27.8	25.7	5.9	6.5	18.4	20.6	33.4	68.9	40.6	62.1
Non-rural provider	37.0	42.8	27.5	25.2	6.3	7.5	22.8	19.8	32.0	56.9	30.2	40.1
Rural provider	39.1	36.1	26.3	24.0	7.4	5.8	22.9	24.8	29.6	26.6	28.6	32.7
English-speaking provider sought	37.5	42.8	27.5	25.2	7.0	7.5	22.5	19.9	32.8	55.3	28.3	38.2
Spanish-speaking provider sought	36.4	38.7	26.5	24.2	2.2	6.6	25.4	21.0	24.3	53.9	41.6	54.3
Carrier A	35.2	36.2	21.1	23.8	0.7	3.6	32.9	24.6	34.6	49.0	20.6	29.0
Carrier B	40.9	42.8	29.4	24.8	6.2	9.2	20.3	19.0	31.9	55.2	26.6	42.7
Carrier C	32.0	43.5	24.4	33.5	1.8	14.5	28.7	11.8	42.5	71.0	26.6	37.9
Carrier D	37.6	45.8	27.5	21.6	6.8	6.2	21.1	19.0	27.2	50.7	23.5	39.1
Carrier E	32.6	41.9	27.7	29.9	15.4	5.8	17.5	14.1	39.0	62.1	45.0	66.0
Carrier F	46.4	44.5	30.5	29.6	3.3	10.8	17.9	16.0	22.4	45.8	27.2	32.2
Carrier G	42.5	39.0	22.6	13.8	2.3	2.2	29.6	36.2	32.3	52.1	32.5	31.0
Carrier H	31.7	37.0	25.8	28.4	1.9	13.2	27.1	18.8	31.2	48.7	93.1	52.2
Carrier I	43.2	26.9	24.9	13.6	2.2	11.6	29.8	47.4	30.0	56.4	19.5	39.5
Carrier J	36.1	41.9	35.6	34.1	9.6	9.1	16.9	14.3	30.6	57.8	28.2	42.3
Carrier K	39.6	43.5	29.5	27.7	6.9	4.4	22.6	23.0	30.2	55.5	27.8	35.8
Carrier L	34.2	49.4	14.3	14.7	6.6	3.1	32.2	24.9	38.2	55.2	32.9	28.8
Carrier M	33.7	40.6	25.9	23.3	7.4	6.7	23.5	22.2	30.0	58.7	33.4	42.9

Abbreviations: ACA, Affordable Care Act; Cardio, cardiologists; Endo, endocrinologists; OLS, ordinary least squares.

Results based on data from a secret-shopper survey of 3740 calls to cardiologists and 3633 calls to endocrinologists for ACA marketplace carriers in Texas. Results are based on OLS or linear probability models with robust SEs. Predicted means are shown. Confidence bounds were omitted. Underlying estimates are included as supplements (Appendices 6–25). Data for 2 ACA insurers were omitted because of the small number of calls. Carriers were blinded. Percentages for success and failure rates may not add up to 100% as some calls identified multiple issues. Outcome definitions and further details are shown in the Appendix 2. Call outcomes were assessed based on the full universe of calls; alternative assessments are based only on fully verified calls in the Appendix 4.

In addition, provider directory errors were encountered in 27.3% of calls (95% CI: 26.1% to 28.5%), ranging from 14.3% to 35.6% across carriers (*P* < .001). The primary type of error was related to contact information (16.8%; 95% CI: 15.8% to 17.8%), with differences ranging from 8.8% to 20.7% across carriers (*P* < .001). Inaccurate specialty listings accounted for 3.9% of errors (95% CI: 3.4% to 4.4%), ranging from 0.1% to 8.8% across carriers (*P* = .001). Callers reaching out to rural providers experienced lower error rates (2.1% vs 4.2%; *P* = .024). Errors related to network status accounted for 3.0% of inaccuracies (95% CI: 2.5% to 3.4%), ranging from 0.5% to 6.3% across carriers (*P* < .001).

Lack of provider capacity was encountered in 6.5% of calls (95% CI: 5.8% to 7.1%), ranging from 1.8% to 15.4% of calls (*P* < .001) across carriers. Moreover, callers seeking Spanish-speaking providers experienced lower capacity issues (2.2% vs 7.0%; *P* < .001).

Due to these challenges, only 22.8% (95% CI: 21.7% to 24.0%) of calls resulted in an appointment. Success rates ranged from 16.9% to 32.2% across carriers (*P* < .001). Successful patients had to wait 31.7 days (95% CI: 29.1 to 34.3 days). Differences across carriers ranged from 22.4 days to 42.5 days (*P* = .044). To reach their provider, cardiology patients had to travel 30.0 minutes (95% CI: 28.0 to 32.0 minutes), with rural patients facing additional travel burdens (40.6 to 26.9 minutes; *P* < .001). Differences across carriers ranged from 19.5 minutes to 93.1 minutes (*P* < .001).

### Endocrinologists

Callers also had difficulty reaching endocrinologists, with 42.5% (95% CI: 41.1% to 43.8%) failing to connect to an individual to verify information. Failure-to-connect rates ranged from 26.9% to 49.4% (*P* = .002) across carriers.

Callers to endocrinologists also encountered provider directory errors in 25.2% of calls (95% CI: 23.8% to 26.6%), ranging from 13.6% to 34.1% across carriers (*P* < .001). Here, contact information errors were encountered in 13.3% of calls (95% CI: 12.2% to 14.4%; ranging from 1.8% to 22.2% across carriers; *P* < .001), inaccurate specialty listings in 3.4% (95% CI: 2.9% to 4.0%; ranging from 0.0% to 10.0% across carriers; *P* < .001), and errors related to network status in 3.6% (95% CI: 3.0% to 4.2%; ranging from 0.0% to 10.5% across carriers; *P* < .001). For endocrinologists, lack of capacity was experienced by 7.4% of callers (95% CI: 6.6% to 8.3%), ranging from 2.2% to 14.5% of calls (*P* < .001) across carriers.

As a result, only 20.0% (95% CI: 18.7% to 21.3%) of calls to endocrinologists resulted in being able to secure an appointment. Success rates ranged from 14.1% to 47.4% across carriers (*P* < .001). Successful endocrinology patients had to wait 55.2 days for care (95% CI: 51.9 to 58.4 days). Differences across carriers ranged from 45.8 days to 71.0 days (*P* < .001). Appointments with rural endocrinologists required an additional 30.3 days (*P* < .001). To seek care, endocrinology patients had to travel 39.7 minutes (95% CI: 36.8 to 42.6 minutes), with rural patients traveling an additional 29.9 minutes (62.1 to 32.2 minutes; *P* < .001). Differences across carriers ranged from 28.8 minutes to 66.0 minutes (*P* < .001).

## Discussion

Texas ACA consumers had difficulty accessing cardiologists and endocrinologists due to directory errors and inability to connect with providers. Given that calls took place during regular business hours based on information directly from an online directory, it seems plausible that at least some of the failed calls may have been inaccurate listings, making our findings a conservative assessment of inaccuracies. Even successful patients often encountered long wait times and may have to travel far. Rural patients faced particular challenges. Callers had more difficulty connecting with endocrinologists (delta: 5.2; *P* < .001) and faced longer wait times (delta: 23.5; *P* < .001) and travel distance (delta: 9.7; *P* < .001).

The findings raise questions about regulatory oversight as well as how well carriers comply with federal and state requirements. The substantial challenges identified suggest that existing monitoring and enforcement mechanisms may need to be re-evaluated. This may include nuanced penalties for excessive or persistent errors. The differences we identified across carriers suggest that carriers approach issues related to provider directories and network adequacy differently and may dedicate differential amounts of resources to support patients. Best practices should be identified and encouraged. Moreover, rural provider networks exhibit particular challenges and may require creative policy solutions. Future work should expand to additional specialties that have seen little attention as well as more ACA markets.

## Supplementary Material

qxag068_Supplementary_Data

## References

[1] Braunwald E . Cardiology: the past, the present, and the future. J Am Coll Cardiol. 2003;42(12):2031–2041. 10.1016/j.jacc.2003.08.025 14680723

[2] Romeo GR, Caputo T, Stanescu IW, Alkhaddo JB. The arduous path toward equitable access to endocrinology care. J Endocr Soc. 2024;8(9):bvae134. 10.1210/jendso/bvae134 39071475 PMC11273240

[3] Burman A, Haeder SF. Potemkin protections: assessing provider directory accuracy and timely access for four specialties in California. J Health Polit Policy Law. 2022;47(3):319–349. 10.1215/03616878-9626866 34847230

[4] Haeder SF, Xu WY, Elton T, Pitcher A. State efforts to regulate provider networks and directories: lessons for the future. J Health Polit Policy Law. 2023;48(6):951–968. 10.1215/03616878-10852610 37497889

[5] KFF . Marketplace Enrollment, 2014-2025. KFF; 2026.

[6] Haeder SF, Weimer DL, Mukamel DB. A consumer-centric approach to network adequacy: access to four specialties in California's marketplace. Health Aff (Milwood). 2019;38(11):1918–1926. 10.1377/hlthaff.2019.00116 31682498

[7] Haeder SF, Xu WY. What methods do patients use to schedule medical appointments? Health Aff Sch. 2025;3(4):qxaf077. 10.1093/haschl/qxaf077 40391103 PMC12086685

[8] Burman A, Haeder SF. Directory accuracy and timely access to in Maryland's Medicaid managed care program. J Health Care Poor Underserved. 2022;33(2):597–611. 10.1353/hpu.2022.0050 35574863

