# Supplementary material for: Assessing access to cardiologists and endocrinologists in the Texas ACA market
Source: Health Aff Sch. 2026 Mar 21;4(4):qxag068. doi: 10.1093/haschl/qxag068 (PMC13044914; doi:10.1093/haschl/qxag068)
Supplement: qxag068_Supplementary_Data [file qxag068_supplementary_data.zip › Appendix 3.docx]

**Appendix 1:** Data Collection Process

We conducted a consumer-centric, large-scale secret shopper survey of in-network providers for across all ACA marketplace carriers in Texas. We distributed patient assignment proportional to ACA marketplace enrollment within the state based on ACA pricing region. For each carrier in each pricing region, we selected the most commonly offered insurance plan. Simulated patients were assigned equally to carriers within each pricing regions.

To collect data, trained callers were assigned a simulated patient as well as relevant information such as a home address, name, non-urgent medical condition and patient age and birth date, and ACA insurance plan. Callers then went to a carrier’s online provider directory and searched a providers based on their home address. Callers then contacted the provider on behalf of a close relative, like a sister or aunt, seeking the first available appointment while documenting their experience. In line with previous research, callers also abandoned calls and moved on to the next provider listed if they were put on hold for more than five minutes. In order avoid wasting medical resources, no actual appointments were scheduled.

**Appendix 2**: Outcome Definitions

| **Outcome** | **Definition** |
| --- | --- |
| Failure to Connect | Callers experienced one of the following:   - Callers only reached a busy signal - Callers only reached an answering machine - Callers only reached a computer system and were unable to connect with a representative - Office was closed |
| Staff Refusal | Callers experienced one of the following:   - Staff required additional information such as medical records before providing any information - Staff refused to talk to anyone but the patient |
| Any Directory Errors | Callers experienced one of the following inaccuracies:   - Calls with Errors Related to Provider Contact Information - Calls with Errors Related to Provider Specialty Information - Calls with Errors Related to Provider Network Status |
| Calls with Errors Related to Provider Contact Information | Callers experienced one of the following problems:   - Provider did not work at the location called - Number called was disconnected - Number called was a fax number - Number called was not a medical office |
| Calls with Errors Related to Provider Specialty Information | Provider did not practice the specialty listed in the provider directory |
| Calls with Errors Related to Provider Network Status | Provider did not accept the insurance plan listed in the provider directory |
| Capacity Limitations | Staff indicated that they either did not accept new patients or schedule any new appointments. |
| Appointments | Callers were able to secure an appointment. |
| Wait Time | Differences between the date of the call and the date of the appointment in days. |
| Travel Time | Distance between the patient’s home address and the provider’s office address. |

**Appendix 3**. Data Overview and Successful Patients

| **Variable** | **Number of Calls** | **Percentage of Call** | **Number of Calls** | **Percentage of Call** |
| --- | --- | --- | --- | --- |
|  | **Cardio** | | **Endo** | |
| Non-Rural Patient | 2,669 | 71.36 | 2,740 | 75.42 |
| Rural Patient | 1,071 | 28.64 | 893 | 24.58 |
| Non-Rural Provider | 3,202 | 85.61 | 3,466 | 95.4 |
| Rural Provider | 538 | 14.39 | 167 | 4.6 |
| English-Speaking Provider Sought | 3,320 | 88.77 | 3,323 | 91.47 |
| Spanish-Speaking Provider Sought | 420 | 11.23 | 310 | 8.53 |
| Carrier A | 251 | 6.71 | 245 | 6.74 |
| Carrier B | 758 | 20.27 | 801 | 22.05 |
| Carrier C | 94 | 2.51 | 179 | 4.93 |
| Carrier D | 717 | 19.17 | 569 | 15.66 |
| Carrier E | 188 | 5.03 | 160 | 4.40 |
| Carrier F | 82 | 2.19 | 71 | 1.95 |
| Carrier G | 75 | 2.01 | 44 | 1.21 |
| Carrier H | 117 | 3.13 | 91 | 2.50 |
| Carrier I | 97 | 2.59 | 51 | 1.40 |
| Carrier J | 389 | 10.4 | 395 | 10.87 |
| Carrier K | 316 | 8.45 | 260 | 7.16 |
| Carrier L | 203 | 5.43 | 225 | 6.19 |
| Carrier M | 436 | 11.66 | 542 | 14.92 |

Notes: Results based on data from a secret shopper survey of 3,740 calls to cardiologists and 3,633 calls to endocrinologists for ACA marketplace carriers in Texas. Results based on OLS or linear probability models with robust standard errors. Predicted means shown. Confidence bounds omitted. Underlying estimates included as supplements. Data for two ACA insurers for were omitted because of the small number of calls. Carriers blinded. Percentages for success and failure rates may not add up to 100% as some calls identified multiple issues.

**Appendix 4**. Overview of Unsuccessful and Successful Calls, Verified Calls Only

| **Variable** | **Any Directory Errors**  **% of Calls** | | **Capacity Limitations**  **% of Calls** | | **Appointments**  **% of Calls** | | **Wait Time to Appointment**  **(Days)** | | **Travel Time (minutes)** | |
| --- | --- | --- | --- | --- | --- | --- | --- | --- | --- | --- |
|  | Cardio | Endo | Cardio | Endo | Cardio | Endo | Cardio | Endo | Cardio | Endo |
| Overall | 43.4 | 42.3 | 12.1 | 15.1 | 42.6 | 40.7 | 31.7 | 55.2 | 30.0 | 39.7 |
| Non-Rural Patient | 41.4 | 42.4 | 12.2 | 15.8 | 44.7 | 40.6 | 31.2 | 50.6 | 26.9 | 32.2 |
| Rural Patient | 49.1 | 41.8 | 11.8 | 13.2 | 36.7 | 41.0 | 33.4 | 68.9 | 40.6 | 62.1 |
| Non-Rural Provider | 43.8 | 42.2 | 11.8 | 15.3 | 42.4 | 40.4 | 32.0 | 56.9 | 30.2 | 40.1 |
| Rural Provider | 41.2 | 43.1 | 14.1 | 11.4 | 44.0 | 45.9 | 29.6 | 26.6 | 28.6 | 32.7 |
| English-Speaking Provider Sought | 43.3 | 42.0 | 13.3 | 15.4 | 42.3 | 40.7 | 32.8 | 55.3 | 28.3 | 38.2 |
| Spanish-Speaking Provider Sought | 44.5 | 45.6 | 3.6 | 12.7 | 44.4 | 41.1 | 24.3 | 53.9 | 41.6 | 54.3 |
| Carrier A | 32.6 | 37.4 | 1.1 | 6.6 | 57.6 | 46.4 | 34.6 | 49.0 | 20.6 | 29.0 |
| Carrier B | 48.0 | 44.7 | 12.3 | 18.8 | 40.5 | 39.0 | 31.9 | 55.2 | 26.6 | 42.7 |
| Carrier C | 35.2 | 59.0 | 3.0 | 28.1 | 54.9 | 22.7 | 42.5 | 71.0 | 26.6 | 37.9 |
| Carrier D | 45.3 | 38.8 | 12.8 | 13.9 | 39.2 | 42.6 | 27.2 | 50.7 | 23.5 | 39.1 |
| Carrier E | 43.5 | 51.6 | 26.0 | 11.3 | 28.7 | 27.8 | 39.0 | 62.1 | 45.0 | 66.0 |
| Carrier F | 49.1 | 38.0 | 7.8 | 26.6 | 42.6 | 38.3 | 22.4 | 45.8 | 27.2 | 32.2 |
| Carrier G | 30.6 | 19.8 | 4.7 | 4.2 | 61.4 | 63.6 | 32.3 | 52.1 | 32.5 | 31.0 |
| Carrier H | 37.4 | 44.1 | 2.7 | 25.0 | 44.4 | 35.7 | 31.2 | 48.7 | 93.1 | 52.2 |
| Carrier I | 35.3 | 21.5 | 4.8 | 17.9 | 60.3 | 73.0 | 30.0 | 56.4 | 19.5 | 39.5 |
| Carrier J | 56.4 | 55.7 | 16.9 | 18.1 | 29.6 | 28.8 | 30.6 | 57.8 | 28.2 | 42.3 |
| Carrier K | 48.0 | 43.6 | 12.5 | 8.4 | 42.0 | 44.0 | 30.2 | 55.5 | 27.8 | 35.8 |
| Carrier L | 24.8 | 27.3 | 13.3 | 7.4 | 64.2 | 58.9 | 38.2 | 55.2 | 32.9 | 28.8 |
| Carrier M | 38.4 | 35.5 | 13.1 | 13.5 | 42.7 | 44.5 | 30.0 | 58.7 | 33.4 | 42.9 |

Notes: Results based on data from a secret shopper survey of 3,740 calls to cardiologists and 3,633 calls to endocrinologists for ACA marketplace carriers in Texas. Results based on OLS or linear probability models with robust standard errors. Predicted means shown. Confidence bounds omitted. Data for two ACA insurers were omitted because of the small number of calls. Carriers blinded. Percentages for success and failure rates may not add up to 100% as some calls identified multiple issues. Call outcomes were assessed based for verified calls only.

**Appendix 5:** Sample Regression Output, Linear Probability Model, Appointment Success, Cardiologists

|  | (1) |
| --- | --- |
| VARIABLES | Appointment Success |
|  |  |
| Rural Caller | -0.063*** |
|  | (0.000) |
| Rural Provider | 0.001 |
|  | (0.978) |
| Spanish-speaking Provider Requested | 0.029 |
|  | (0.207) |
| Carrier B | -0.126*** |
|  | (0.000) |
| Carrier C | -0.042 |
|  | (0.444) |
| Carrier D | -0.118*** |
|  | (0.000) |
| Carrier E | -0.154*** |
|  | (0.000) |
| Carrier F | -0.150** |
|  | (0.005) |
| Carrier G | -0.033 |
|  | (0.585) |
| Carrier H | -0.058 |
|  | (0.259) |
| Carrier I | -0.030 |
|  | (0.581) |
| Carrier J | -0.160*** |
|  | (0.000) |
| Carrier K | -0.103** |
|  | (0.007) |
| Carrier L | -0.007 |
|  | (0.882) |
| Carrier M | -0.094** |
|  | (0.009) |
| Constant | 0.344*** |
|  | (0.000) |
|  |  |
| Observations | 3,723 |
| R-squared | 0.018 |

Robust p-values in parentheses

*** p<0.001, ** p<0.01, * p<0.05

**Appendix 6:** Percentage of Calls Unable to Verify for Failure to Connect, Predicted Means, Cardiologists

| **Variable** | **Variable** | **95% Confidence Bounds** | |
| --- | --- | --- | --- |
| Non-Rural Patient | 42.5% | 40.6% | 44.4% |
| Rural Patient | 42.4% | 39.1% | 45.8% |
| Non-Rural Provider | 42.8% | 41.1% | 44.4% |
| Rural Provider | 36.1% | 28.6% | 43.7% |
| English-Speaking Provider Sought | 42.8% | 41.1% | 44.5% |
| Spanish-Speaking Provider Sought | 38.7% | 33.2% | 44.2% |
| Carrier A | 36.2% | 30.1% | 42.2% |
| Carrier B | 42.8% | 39.4% | 46.3% |
| Carrier C | 43.5% | 36.3% | 50.8% |
| Carrier D | 45.8% | 41.7% | 49.9% |
| Carrier E | 41.9% | 34.2% | 49.6% |
| Carrier F | 44.5% | 32.8% | 56.1% |
| Carrier G | 39.0% | 24.7% | 53.2% |
| Carrier H | 37.0% | 27.0% | 47.0% |
| Carrier I | 26.9% | 14.6% | 39.2% |
| Carrier J | 41.9% | 37.0% | 46.8% |
| Carrier K | 43.5% | 37.5% | 49.6% |
| Carrier L | 49.4% | 42.8% | 55.9% |
| Carrier M | 40.6% | 36.4% | 44.7% |

Notes: Results based on data from a secret shopper survey of 3,740 calls to cardiologists for ACA marketplace carriers in Texas. Results based on linear probability models with robust standard errors. Predicted means shown. Data for two ACA insurers were omitted because of the small number of calls. Carriers blinded.

**Appendix 7:** Percentage of Calls Unable to Verify for Failure to Connect, Predicted Means, Endocrinologists

| **Variable** | **Variable** | **95% Confidence Bounds** | |
| --- | --- | --- | --- |
| Non-Rural Patient | 42.5% | 40.6% | 44.4% |
| Rural Patient | 42.4% | 39.1% | 45.8% |
| Non-Rural Provider | 42.8% | 41.1% | 44.4% |
| Rural Provider | 36.1% | 28.6% | 43.7% |
| English-Speaking Provider Sought | 42.8% | 41.1% | 44.5% |
| Spanish-Speaking Provider Sought | 38.7% | 33.2% | 44.2% |
| Carrier A | 36.2% | 30.1% | 42.2% |
| Carrier B | 42.8% | 39.4% | 46.3% |
| Carrier C | 43.5% | 36.3% | 50.8% |
| Carrier D | 45.8% | 41.7% | 49.9% |
| Carrier E | 41.9% | 34.2% | 49.6% |
| Carrier F | 44.5% | 32.8% | 56.1% |
| Carrier G | 39.0% | 24.7% | 53.2% |
| Carrier H | 37.0% | 27.0% | 47.0% |
| Carrier I | 26.9% | 14.6% | 39.2% |
| Carrier J | 41.9% | 37.0% | 46.8% |
| Carrier K | 43.5% | 37.5% | 49.6% |
| Carrier L | 49.4% | 42.8% | 55.9% |
| Carrier M | 40.6% | 36.4% | 44.7% |

Notes: Results based on data from a secret shopper survey of 3,633 calls to endocrinologists for ACA marketplace carriers in Texas. Results based on linear probability models with robust standard errors. Predicted means shown. Data for two ACA insurers were omitted because of the small number of calls. Carriers blinded.

**Appendix 8:** Percentage of Calls with Staff Refusing to Provide Information, Predicted Means, Cardiologists

| **Variable** | **Variable** | **95% Confidence Bounds** | |
| --- | --- | --- | --- |
| Non-Rural Patient | 4.4% | 3.6% | 5.1% |
| Rural Patient | 5.5% | 4.1% | 6.8% |
| Non-Rural Provider | 4.7% | 4.0% | 5.4% |
| Rural Provider | 4.5% | 2.6% | 6.5% |
| English-Speaking Provider Sought | 4.7% | 4.0% | 5.5% |
| Spanish-Speaking Provider Sought | 4.2% | 2.3% | 6.0% |
| Carrier A | 5.3% | 2.5% | 8.0% |
| Carrier B | 3.6% | 2.3% | 5.0% |
| Carrier C | 7.5% | 2.1% | 12.8% |
| Carrier D | 5.5% | 3.8% | 7.2% |
| Carrier E | 3.0% | 0.5% | 5.6% |
| Carrier F | 6.4% | 1.2% | 11.6% |
| Carrier G | 6.6% | 1.0% | 12.3% |
| Carrier H | 3.3% | 0.1% | 6.6% |
| Carrier I | 5.2% | 0.8% | 9.6% |
| Carrier J | 1.9% | 0.5% | 3.2% |
| Carrier K | 2.0% | 0.5% | 3.5% |
| Carrier L | 11.9% | 7.4% | 16.3% |
| Carrier M | 5.5% | 3.3% | 7.6% |

Notes: Results based on data from a secret shopper survey of 3,740 calls to cardiologists for ACA marketplace carriers in Texas. Results based on linear probability models with robust standard errors. Predicted means shown. Data for two ACA insurers were omitted because of the small number of calls. Carriers blinded.

**Appendix 9:** Percentage of Calls with Staff Refusing to Provide Information, Predicted Means, Endocrinologists

| **Variable** | **Variable** | **95% Confidence Bounds** | |
| --- | --- | --- | --- |
| Non-Rural Patient | 4.6% | 3.9% | 5.4% |
| Rural Patient | 4.1% | 2.8% | 5.4% |
| Non-Rural Provider | 4.5% | 3.8% | 5.1% |
| Rural Provider | 5.8% | 2.2% | 9.4% |
| English-Speaking Provider Sought | 4.3% | 3.6% | 5.0% |
| Spanish-Speaking Provider Sought | 6.8% | 4.0% | 9.6% |
| Carrier A | 4.6% | 1.8% | 7.3% |
| Carrier B | 4.0% | 2.6% | 5.3% |
| Carrier C | 1.1% | -0.5% | 2.7% |
| Carrier D | 5.5% | 3.6% | 7.4% |
| Carrier E | 3.3% | 0.6% | 6.0% |
| Carrier F | 5.7% | 0.4% | 11.1% |
| Carrier G | 2.1% | -2.2% | 6.5% |
| Carrier H | 6.8% | 1.7% | 11.9% |
| Carrier I | 4.2% | -1.2% | 9.5% |
| Carrier J | 3.7% | 1.9% | 5.5% |
| Carrier K | 0.8% | -0.3% | 1.9% |
| Carrier L | 6.6% | 3.4% | 9.9% |
| Carrier M | 7.0% | 4.8% | 9.1% |

Notes: Results based on data from a secret shopper survey of 3,633 calls to endocrinologists for ACA marketplace carriers in Texas. Results based on linear probability models with robust standard errors. Predicted means shown. Data for two ACA insurers were omitted because of the small number of calls. Carriers blinded.

**Appendix 10:** Percentage of Calls with Any Provider Directory Errors, Predicted Means, Cardiologists

| **Variable** | **Variable** | **95% Confidence Bounds** | |
| --- | --- | --- | --- |
| Non-Rural Patient | 27.2% | 25.4% | 28.9% |
| Rural Patient | 27.8% | 24.9% | 30.7% |
| Non-Rural Provider | 27.5% | 26.0% | 29.1% |
| Rural Provider | 26.3% | 22.2% | 30.4% |
| English-Speaking Provider Sought | 27.5% | 25.9% | 29.0% |
| Spanish-Speaking Provider Sought | 26.5% | 22.3% | 30.7% |
| Carrier A | 21.1% | 16.0% | 26.1% |
| Carrier B | 29.4% | 26.1% | 32.6% |
| Carrier C | 24.4% | 15.7% | 33.1% |
| Carrier D | 27.5% | 24.3% | 30.8% |
| Carrier E | 27.7% | 21.3% | 34.2% |
| Carrier F | 30.5% | 20.4% | 40.5% |
| Carrier G | 22.6% | 13.1% | 32.2% |
| Carrier H | 25.8% | 17.9% | 33.8% |
| Carrier I | 24.9% | 16.2% | 33.5% |
| Carrier J | 35.6% | 30.8% | 40.4% |
| Carrier K | 29.5% | 24.4% | 34.5% |
| Carrier L | 14.3% | 9.4% | 19.1% |
| Carrier M | 25.9% | 21.8% | 30.0% |

Notes: Results based on data from a secret shopper survey of 3,740 calls to cardiologists for ACA marketplace carriers in Texas. Results based on linear probability models with robust standard errors. Predicted means shown. Data for two ACA insurers were omitted because of the small number of calls. Carriers blinded.

**Appendix 11:** Percentage of Calls with Any Provider Directory Errors, Predicted Means, Endocrinologists

| **Variable** | **Variable** | **95% Confidence Bounds** | |
| --- | --- | --- | --- |
| Non-Rural Patient | 25.0% | 23.4% | 26.6% |
| Rural Patient | 25.7% | 22.7% | 28.7% |
| Non-Rural Provider | 25.2% | 23.8% | 26.7% |
| Rural Provider | 24.0% | 17.5% | 30.6% |
| English-Speaking Provider Sought | 25.2% | 23.8% | 26.7% |
| Spanish-Speaking Provider Sought | 24.2% | 19.4% | 29.0% |
| Carrier A | 23.8% | 18.4% | 29.2% |
| Carrier B | 24.8% | 21.8% | 27.8% |
| Carrier C | 33.5% | 26.6% | 40.4% |
| Carrier D | 21.6% | 18.2% | 25.0% |
| Carrier E | 29.9% | 22.8% | 37.0% |
| Carrier F | 29.6% | 18.9% | 40.3% |
| Carrier G | 13.8% | 3.6% | 24.0% |
| Carrier H | 28.4% | 19.1% | 37.7% |
| Carrier I | 13.6% | 4.1% | 23.1% |
| Carrier J | 34.1% | 29.3% | 38.8% |
| Carrier K | 27.7% | 22.3% | 33.2% |
| Carrier L | 14.7% | 10.1% | 19.3% |
| Carrier M | 23.3% | 19.7% | 26.8% |

Notes: Results based on data from a secret shopper survey of 3,633 calls to endocrinologists for ACA marketplace carriers in Texas. Results based on linear probability models with robust standard errors. Predicted means shown. Data for two ACA insurers were omitted because of the small number of calls. Carriers blinded.

**Appendix 12:** Percentage of Calls with Errors Related to Provider Contact Information, Predicted Means, Cardiologists

| **Variable** | **Variable** | **95% Confidence Bounds** | |
| --- | --- | --- | --- |
| Non-Rural Patient | 16.8% | 15.3% | 18.2% |
| Rural Patient | 16.9% | 14.5% | 19.3% |
| Non-Rural Provider | 16.9% | 15.6% | 18.2% |
| Rural Provider | 16.1% | 12.7% | 19.5% |
| English-Speaking Provider Sought | 16.8% | 15.5% | 18.0% |
| Spanish-Speaking Provider Sought | 17.1% | 13.5% | 20.7% |
| Carrier A | 9.5% | 5.9% | 13.2% |
| Carrier B | 16.4% | 13.7% | 19.0% |
| Carrier C | 14.9% | 7.7% | 22.1% |
| Carrier D | 20.7% | 17.7% | 23.7% |
| Carrier E | 11.2% | 6.7% | 15.8% |
| Carrier F | 20.7% | 11.8% | 29.5% |
| Carrier G | 18.7% | 9.9% | 27.6% |
| Carrier H | 13.8% | 7.5% | 20.0% |
| Carrier I | 14.5% | 7.5% | 21.5% |
| Carrier J | 20.7% | 16.6% | 24.8% |
| Carrier K | 15.8% | 11.7% | 19.8% |
| Carrier L | 8.8% | 4.9% | 12.8% |
| Carrier M | 19.3% | 15.6% | 23.0% |

Notes: Results based on data from a secret shopper survey of 3,740 calls to cardiologists for ACA marketplace carriers in Texas. Results based on linear probability models with robust standard errors. Predicted means shown. Data for two ACA insurers were omitted because of the small number of calls. Carriers blinded.

**Appendix 13:** Percentage of Calls with Errors Related to Provider Contact Information, Predicted Means, Endocrinologists

| **Variable** | **Variable** | **95% Confidence Bounds** | |
| --- | --- | --- | --- |
| Non-Rural Patient | 13.5% | 12.2% | 14.7% |
| Rural Patient | 12.9% | 10.6% | 15.2% |
| Non-Rural Provider | 13.4% | 12.2% | 14.5% |
| Rural Provider | 12.3% | 7.3% | 17.3% |
| English-Speaking Provider Sought | 13.5% | 12.3% | 14.6% |
| Spanish-Speaking Provider Sought | 11.8% | 8.2% | 15.3% |
| Carrier A | 9.9% | 6.1% | 13.7% |
| Carrier B | 10.4% | 8.3% | 12.6% |
| Carrier C | 12.3% | 7.5% | 17.1% |
| Carrier D | 13.9% | 11.0% | 16.7% |
| Carrier E | 14.4% | 8.9% | 19.8% |
| Carrier F | 22.2% | 12.5% | 32.0% |
| Carrier G | 6.8% | -0.6% | 14.3% |
| Carrier H | 13.2% | 6.2% | 20.1% |
| Carrier I | 1.8% | -2.0% | 5.6% |
| Carrier J | 21.5% | 17.4% | 25.5% |
| Carrier K | 16.0% | 11.5% | 20.5% |
| Carrier L | 8.9% | 5.2% | 12.6% |
| Carrier M | 13.7% | 10.8% | 16.6% |

Notes: Results based on data from a secret shopper survey of 3,633 calls to endocrinologists for ACA marketplace carriers in Texas. Results based on linear probability models with robust standard errors. Predicted means shown. Data for two ACA insurers were omitted because of the small number of calls. Carriers blinded.

**Appendix 14:** Percentage of Calls with Errors Related to Provider Specialty Information, Predicted Means, Cardiologists

| **Variable** | **Variable** | **95% Confidence Bounds** | |
| --- | --- | --- | --- |
| Non-Rural Patient | 3.7% | 3.0% | 4.5% |
| Rural Patient | 4.4% | 3.1% | 5.7% |
| Non-Rural Provider | 4.2% | 3.5% | 4.9% |
| Rural Provider | 2.1% | 0.5% | 3.7% |
| English-Speaking Provider Sought | 3.9% | 3.2% | 4.5% |
| Spanish-Speaking Provider Sought | 4.5% | 2.5% | 6.4% |
| Carrier A | 5.1% | 2.4% | 7.8% |
| Carrier B | 4.5% | 3.0% | 5.9% |
| Carrier C | 4.3% | 0.3% | 8.4% |
| Carrier D | 3.0% | 1.8% | 4.3% |
| Carrier E | 8.1% | 4.2% | 12.0% |
| Carrier F | 3.6% | -0.5% | 7.7% |
| Carrier G | 0.1% | -0.1% | 0.4% |
| Carrier H | 8.8% | 3.7% | 13.9% |
| Carrier I | 5.3% | 0.8% | 9.7% |
| Carrier J | 4.9% | 2.7% | 7.1% |
| Carrier K | 2.8% | 0.9% | 4.6% |
| Carrier L | 0.9% | -0.5% | 2.3% |
| Carrier M | 2.3% | 0.9% | 3.8% |

Notes: Results based on data from a secret shopper survey of 3,740 calls to cardiologists for ACA marketplace carriers in Texas. Results based on linear probability models with robust standard errors. Predicted means shown. Data for two ACA insurers were omitted because of the small number of calls. Carriers blinded.

**Appendix 15:** Percentage of Calls with Errors Related to Provider Specialty Information, Predicted Means, Endocrinologists

| **Variable** | **Variable** | **95% Confidence Bounds** | |
| --- | --- | --- | --- |
| Non-Rural Patient | 3.4% | 2.7% | 4.1% |
| Rural Patient | 3.5% | 2.2% | 4.7% |
| Non-Rural Provider | 3.4% | 2.8% | 4.0% |
| Rural Provider | 4.2% | 1.2% | 7.1% |
| English-Speaking Provider Sought | 3.3% | 2.7% | 4.0% |
| Spanish-Speaking Provider Sought | 4.5% | 2.1% | 6.9% |
| Carrier A | 7.6% | 4.3% | 11.0% |
| Carrier B | 4.1% | 2.7% | 5.5% |
| Carrier C | 10.0% | 5.6% | 14.5% |
| Carrier D | 1.2% | 0.3% | 2.1% |
| Carrier E | 3.8% | 0.8% | 6.8% |
| Carrier F | 0.1% | -0.3% | 0.5% |
| Carrier G | 0.0% | -0.3% | 0.2% |
| Carrier H | 2.3% | -0.8% | 5.3% |
| Carrier I | 0.1% | -0.1% | 0.3% |
| Carrier J | 4.4% | 2.3% | 6.4% |
| Carrier K | 2.0% | 0.3% | 3.6% |
| Carrier L | 1.8% | 0.0% | 3.5% |
| Carrier M | 2.6% | 1.2% | 3.9% |

Notes: Results based on data from a secret shopper survey of 3,633 calls to endocrinologists for ACA marketplace carriers in Texas. Results based on linear probability models with robust standard errors. Predicted means shown. Data for two ACA insurers were omitted because of the small number of calls. Carriers blinded.

**Appendix 16:** Percentage of Calls with Errors Related to Provider Network Status, Predicted Means, Cardiologists

| **Variable** | **Variable** | **95% Confidence Bounds** | |
| --- | --- | --- | --- |
| Non-Rural Patient | 3.2% | 2.5% | 3.9% |
| Rural Patient | 2.4% | 1.5% | 3.2% |
| Non-Rural Provider | 3.0% | 2.5% | 3.6% |
| Rural Provider | 2.5% | 1.3% | 3.6% |
| English-Speaking Provider Sought | 3.1% | 2.5% | 3.7% |
| Spanish-Speaking Provider Sought | 2.1% | 0.7% | 3.5% |
| Carrier A | 6.2% | 3.2% | 9.2% |
| Carrier B | 4.5% | 3.0% | 6.0% |
| Carrier C | 1.0% | -1.1% | 3.0% |
| Carrier D | 0.8% | 0.2% | 1.4% |
| Carrier E | 1.3% | -0.2% | 2.8% |
| Carrier F | 3.3% | -0.8% | 7.4% |
| Carrier G | 2.6% | -1.0% | 6.3% |
| Carrier H | 2.8% | -0.1% | 5.7% |
| Carrier I | 2.1% | -0.7% | 5.0% |
| Carrier J | 4.2% | 2.2% | 6.3% |
| Carrier K | 6.3% | 3.6% | 9.0% |
| Carrier L | 0.5% | -0.5% | 1.5% |
| Carrier M | 0.9% | 0.0% | 1.8% |

Notes: Results based on data from a secret shopper survey of 3,740 calls to cardiologists for ACA marketplace carriers in Texas. Results based on linear probability models with robust standard errors. Predicted means shown. Data for two ACA insurers were omitted because of the small number of calls. Carriers blinded.

**Appendix 17:** Percentage of Calls with Errors Related to Provider Network Status, Predicted Means, Endocrinologists

| **Variable** | **Variable** | **95% Confidence Bounds** | |
| --- | --- | --- | --- |
| Non-Rural Patient | 3.4% | 2.7% | 4.1% |
| Rural Patient | 4.3% | 3.0% | 5.6% |
| Non-Rural Provider | 3.6% | 3.0% | 4.2% |
| Rural Provider | 3.5% | 0.6% | 6.4% |
| English-Speaking Provider Sought | 3.6% | 3.0% | 4.3% |
| Spanish-Speaking Provider Sought | 3.4% | 1.4% | 5.5% |
| Carrier A | 4.6% | 2.0% | 7.3% |
| Carrier B | 3.7% | 2.4% | 5.0% |
| Carrier C | 0.0% | -0.1% | 0.1% |
| Carrier D | 1.9% | 0.8% | 3.1% |
| Carrier E | 10.5% | 5.8% | 15.3% |
| Carrier F | 3.0% | -0.9% | 6.9% |
| Carrier G | 2.4% | -2.0% | 6.8% |
| Carrier H | 0.0% | -0.4% | 0.1% |
| Carrier I | 0.0% | -0.3% | 0.2% |
| Carrier J | 5.5% | 3.2% | 7.8% |
| Carrier K | 7.8% | 4.6% | 11.0% |
| Carrier L | 1.4% | -0.1% | 2.9% |
| Carrier M | 2.6% | 1.2% | 3.9% |

Notes: Results based on data from a secret shopper survey of 3,633 calls to endocrinologists for ACA marketplace carriers in Texas. Results based on linear probability models with robust standard errors. Predicted means shown. Data for two ACA insurers were omitted because of the small number of calls. Carriers blinded.

**Appendix 18:** Percentage of Calls with Capacity Limitations, Predicted Means, Cardiologists

| **Variable** | **Variable** | **95% Confidence Bounds** | |
| --- | --- | --- | --- |
| Non-Rural Patient | 6.7% | 5.7% | 7.7% |
| Rural Patient | 5.9% | 4.2% | 7.7% |
| Non-Rural Provider | 6.3% | 5.4% | 7.2% |
| Rural Provider | 7.4% | 4.8% | 10.0% |
| English-Speaking Provider Sought | 7.0% | 6.1% | 7.9% |
| Spanish-Speaking Provider Sought | 2.2% | 0.8% | 3.7% |
| Carrier A | 0.7% | -0.4% | 1.8% |
| Carrier B | 6.2% | 4.5% | 7.9% |
| Carrier C | 1.8% | -1.1% | 4.7% |
| Carrier D | 6.8% | 4.9% | 8.6% |
| Carrier E | 15.4% | 10.2% | 20.6% |
| Carrier F | 3.3% | -0.8% | 7.4% |
| Carrier G | 2.3% | -1.4% | 5.9% |
| Carrier H | 1.9% | -0.5% | 4.2% |
| Carrier I | 2.2% | -0.6% | 5.0% |
| Carrier J | 9.6% | 6.7% | 12.5% |
| Carrier K | 6.9% | 4.1% | 9.6% |
| Carrier L | 6.6% | 3.2% | 10.0% |
| Carrier M | 7.4% | 4.9% | 9.8% |

Notes: Results based on data from a secret shopper survey of 3,740 calls to cardiologists for ACA marketplace carriers in Texas. Results based on linear probability models with robust standard errors. Predicted means shown. Data for two ACA insurers were omitted because of the small number of calls. Carriers blinded.

**Appendix 19:** Percentage of Calls with Capacity Limitations, Predicted Means, Endocrinologists

| **Variable** | **Variable** | **95% Confidence Bounds** | |
| --- | --- | --- | --- |
| Non-Rural Patient | 7.7% | 6.7% | 8.7% |
| Rural Patient | 6.5% | 4.8% | 8.2% |
| Non-Rural Provider | 7.5% | 6.6% | 8.4% |
| Rural Provider | 5.8% | 2.3% | 9.2% |
| English-Speaking Provider Sought | 7.5% | 6.6% | 8.4% |
| Spanish-Speaking Provider Sought | 6.6% | 3.8% | 9.3% |
| Carrier A | 3.6% | 1.3% | 5.9% |
| Carrier B | 9.2% | 7.2% | 11.2% |
| Carrier C | 14.5% | 9.4% | 19.7% |
| Carrier D | 6.2% | 4.2% | 8.2% |
| Carrier E | 5.8% | 2.2% | 9.4% |
| Carrier F | 10.8% | 3.4% | 18.2% |
| Carrier G | 2.2% | -2.2% | 6.7% |
| Carrier H | 13.2% | 6.3% | 20.2% |
| Carrier I | 11.6% | 2.8% | 20.5% |
| Carrier J | 9.1% | 6.2% | 11.9% |
| Carrier K | 4.4% | 1.8% | 6.9% |
| Carrier L | 3.1% | 0.8% | 5.4% |
| Carrier M | 6.7% | 4.6% | 8.8% |

Notes: Results based on data from a secret shopper survey of 3,633 calls to endocrinologists for ACA marketplace carriers in Texas. Results based on linear probability models with robust standard errors. Predicted means shown. Data for two ACA insurers were omitted because of the small number of calls. Carriers blinded.

**Appendix 20:** Call Success Rates, Predicted Means, Cardiologists

| **Variable** | **Variable** | **95% Confidence Bounds** | |
| --- | --- | --- | --- |
| Non-Rural Patient | 24.6% | 23.0% | 26.3% |
| Rural Patient | 18.4% | 15.9% | 20.8% |
| Non-Rural Provider | 22.8% | 21.4% | 24.3% |
| Rural Provider | 22.9% | 19.1% | 26.6% |
| English-Speaking Provider Sought | 22.5% | 21.1% | 23.9% |
| Spanish-Speaking Provider Sought | 25.4% | 21.2% | 29.6% |
| Carrier A | 32.9% | 27.1% | 38.7% |
| Carrier B | 20.3% | 17.4% | 23.2% |
| Carrier C | 28.7% | 19.5% | 37.8% |
| Carrier D | 21.1% | 18.1% | 24.1% |
| Carrier E | 17.5% | 12.2% | 22.9% |
| Carrier F | 17.9% | 9.2% | 26.6% |
| Carrier G | 29.6% | 19.2% | 40.0% |
| Carrier H | 27.1% | 19.0% | 35.2% |
| Carrier I | 29.8% | 20.8% | 38.9% |
| Carrier J | 16.9% | 13.1% | 20.7% |
| Carrier K | 22.6% | 17.9% | 27.2% |
| Carrier L | 32.2% | 25.8% | 38.7% |
| Carrier M | 23.5% | 19.5% | 27.5% |

Notes: Results based on data from a secret shopper survey of 3,740 calls to cardiologists for ACA marketplace carriers in Texas. Results based on linear probability models with robust standard errors. Predicted means shown. Data for two ACA insurers were omitted because of the small number of calls. Carriers blinded.

**Appendix 21:** Call Success Rates, Predicted Means, Endocrinologists

| **Variable** | **Variable** | **95% Confidence Bounds** | |
| --- | --- | --- | --- |
| Non-Rural Patient | 19.8% | 18.3% | 21.3% |
| Rural Patient | 20.6% | 18.0% | 23.3% |
| Non-Rural Provider | 19.8% | 18.4% | 21.1% |
| Rural Provider | 24.8% | 18.2% | 31.4% |
| English-Speaking Provider Sought | 19.9% | 18.5% | 21.2% |
| Spanish-Speaking Provider Sought | 21.0% | 16.3% | 25.7% |
| Carrier A | 24.6% | 19.2% | 30.1% |
| Carrier B | 19.0% | 16.3% | 21.8% |
| Carrier C | 11.8% | 7.1% | 16.5% |
| Carrier D | 19.0% | 15.8% | 22.3% |
| Carrier E | 14.1% | 8.7% | 19.6% |
| Carrier F | 16.0% | 7.5% | 24.5% |
| Carrier G | 36.2% | 21.9% | 50.5% |
| Carrier H | 18.8% | 10.7% | 26.8% |
| Carrier I | 47.4% | 33.6% | 61.1% |
| Carrier J | 14.3% | 10.8% | 17.8% |
| Carrier K | 23.0% | 17.9% | 28.1% |
| Carrier L | 24.9% | 19.2% | 30.5% |
| Carrier M | 22.2% | 18.7% | 25.7% |

Notes: Results based on data from a secret shopper survey of 3,633 calls to endocrinologists for ACA marketplace carriers in Texas. Results based on linear probability models with robust standard errors. Predicted means shown. Data for two ACA insurers were omitted because of the small number of calls. Carriers blinded.

**Appendix 22:** Wait Time to Appointment in Days, Predicted Means, Cardiologists

| **Variable** | **Variable** | **95% Confidence Bounds** | |
| --- | --- | --- | --- |
| Non-Rural Patient | 31.2 | 28.7 | 33.7 |
| Rural Patient | 33.4 | 27.2 | 39.7 |
| Non-Rural Provider | 32.0 | 29.4 | 34.6 |
| Rural Provider | 29.6 | 23.0 | 36.2 |
| English-Speaking Provider Sought | 32.8 | 30.3 | 35.2 |
| Spanish-Speaking Provider Sought | 24.3 | 20.6 | 28.0 |
| Carrier A | 34.6 | 29.2 | 40.0 |
| Carrier B | 31.9 | 26.3 | 37.5 |
| Carrier C | 42.5 | 32.1 | 52.9 |
| Carrier D | 27.2 | 23.7 | 30.7 |
| Carrier E | 39.0 | 19.0 | 58.9 |
| Carrier F | 22.4 | 9.7 | 35.1 |
| Carrier G | 32.3 | 15.1 | 49.5 |
| Carrier H | 31.2 | 23.1 | 39.3 |
| Carrier I | 30.0 | 22.4 | 37.6 |
| Carrier J | 30.6 | 19.7 | 41.5 |
| Carrier K | 30.2 | 24.3 | 36.1 |
| Carrier L | 38.2 | 31.3 | 45.1 |
| Carrier M | 30.0 | 23.0 | 36.9 |

Notes: Results based on data from a secret shopper survey of 3,740 calls to cardiologists for ACA marketplace carriers in Texas. Results based on OLS models with robust standard errors. Predicted means shown. Data for two ACA insurers were omitted because of the small number of calls. Carriers blinded.

**Appendix 23:** Wait Time to Appointment in Days, Predicted Means, Endocrinologists

| **Variable** | **Variable** | **95% Confidence Bounds** | |
| --- | --- | --- | --- |
| Non-Rural Patient | 50.6 | 46.9 | 54.3 |
| Rural Patient | 68.9 | 61.0 | 76.8 |
| Non-Rural Provider | 56.9 | 53.5 | 60.4 |
| Rural Provider | 26.6 | 12.8 | 40.5 |
| English-Speaking Provider Sought | 55.3 | 51.8 | 58.7 |
| Spanish-Speaking Provider Sought | 53.9 | 43.0 | 64.7 |
| Carrier A | 49.0 | 38.6 | 59.5 |
| Carrier B | 55.2 | 47.4 | 63.1 |
| Carrier C | 71.0 | 48.7 | 93.4 |
| Carrier D | 50.7 | 43.3 | 58.0 |
| Carrier E | 62.1 | 41.1 | 83.0 |
| Carrier F | 45.8 | 25.6 | 66.1 |
| Carrier G | 52.1 | 34.5 | 69.6 |
| Carrier H | 48.7 | 28.9 | 68.5 |
| Carrier I | 56.4 | 40.6 | 72.2 |
| Carrier J | 57.8 | 44.2 | 71.4 |
| Carrier K | 55.5 | 46.4 | 64.5 |
| Carrier L | 55.2 | 42.1 | 68.4 |
| Carrier M | 58.7 | 50.3 | 67.0 |

Notes: Results based on data from a secret shopper survey of 3,633 calls to endocrinologists for ACA marketplace carriers in Texas. Results based on OLS models with robust standard errors. Predicted means shown. Data for two ACA insurers were omitted because of the small number of calls. Carriers blinded.

**Appendix 24:** Travel Time to Appointment in Minutes, Predicted Means, Cardiologists

| **Variable** | **Variable** | **95% Confidence Bounds** | |
| --- | --- | --- | --- |
| Non-Rural Patient | 26.9 | 24.0 | 29.7 |
| Rural Patient | 40.6 | 36.0 | 45.3 |
| Non-Rural Provider | 30.2 | 28.0 | 32.4 |
| Rural Provider | 28.6 | 22.2 | 35.0 |
| English-Speaking Provider Sought | 28.3 | 26.2 | 30.4 |
| Spanish-Speaking Provider Sought | 41.6 | 34.5 | 48.7 |
| Carrier A | 20.6 | 18.0 | 23.1 |
| Carrier B | 26.6 | 23.0 | 30.2 |
| Carrier C | 26.6 | 21.2 | 32.0 |
| Carrier D | 23.5 | 21.2 | 25.7 |
| Carrier E | 45.0 | 30.7 | 59.4 |
| Carrier F | 27.2 | 21.8 | 32.6 |
| Carrier G | 32.5 | 26.5 | 38.5 |
| Carrier H | 93.1 | 58.8 | 127.5 |
| Carrier I | 19.5 | 15.7 | 23.3 |
| Carrier J | 28.2 | 22.7 | 33.6 |
| Carrier K | 27.8 | 21.7 | 33.9 |
| Carrier L | 32.9 | 23.7 | 42.1 |
| Carrier M | 33.4 | 26.6 | 40.1 |

Notes: Results based on data from a secret shopper survey of 3,740 calls to cardiologists for ACA marketplace carriers in Texas. Results based on OLS models with robust standard errors. Predicted means shown. Data for two ACA insurers were omitted because of the small number of calls. Carriers blinded.

**Appendix 25:** Travel Time to Appointment in Minutes, Predicted Means, Endocrinologists

| **Variable** | **Variable** | **95% Confidence Bounds** | |
| --- | --- | --- | --- |
| Non-Rural Patient | 32.2 | 28.5 | 35.9 |
| Rural Patient | 62.1 | 57.0 | 67.2 |
| Non-Rural Provider | 40.1 | 37.1 | 43.1 |
| Rural Provider | 32.7 | 19.6 | 45.8 |
| English-Speaking Provider Sought | 38.2 | 35.3 | 41.1 |
| Spanish-Speaking Provider Sought | 54.3 | 41.9 | 66.7 |
| Carrier A | 29.0 | 24.0 | 33.9 |
| Carrier B | 42.7 | 36.8 | 48.5 |
| Carrier C | 37.9 | 18.2 | 57.5 |
| Carrier D | 39.1 | 31.0 | 47.2 |
| Carrier E | 66.0 | 46.3 | 85.7 |
| Carrier F | 32.2 | 24.9 | 39.4 |
| Carrier G | 31.0 | 23.2 | 38.9 |
| Carrier H | 52.2 | 35.6 | 68.8 |
| Carrier I | 39.5 | 25.7 | 53.3 |
| Carrier J | 42.3 | 31.9 | 52.7 |
| Carrier K | 35.8 | 26.4 | 45.2 |
| Carrier L | 28.8 | 23.7 | 33.9 |
| Carrier M | 42.9 | 32.9 | 52.9 |

Notes: Results based on data from a secret shopper survey of 3,633 calls to endocrinologists for ACA marketplace carriers in Texas. Results based on OLS models with robust standard errors. Predicted means shown. Data for two ACA insurers were omitted because of the small number of calls. Carriers blinded.
